# Complete Genome Sequence of a Tilapia Lake Virus Isolate Obtained from Nile Tilapia (Oreochromis niloticus)

**DOI:** 10.1128/genomeA.00580-18

**Published:** 2018-06-28

**Authors:** Lowia Al-Hussinee, Kuttichantran Subramaniam, Mohammad Shamim Ahasan, Bill Keleher, Thomas B. Waltzek

**Affiliations:** a Department of Infectious Disease and Immunology, College of Veterinary Medicine, University of Florida, Gainesville, Florida, USA; b Faculty of Veterinary and Animal Sciences, Hajee Mohammad Danesh Science and Technology University, Dinajpur, Bangladesh; c Kennebec River Biosciences, Richmond, Maine, USA

## Abstract

Since its discovery in 2014, tilapia lake virus (TiLV) has emerged as a significant cause of mortality in tilapia cultured in Asia, Africa, and South America. Here, we report the complete genome sequence of a TiLV isolate obtained during a diagnostic investigation of an ongoing mortality event involving Nile tilapia cultured in Thailand.

## GENOME ANNOUNCEMENT

Tilapia is one of the important aquaculture species and a primary source of protein in many developing countries. In 2016, the estimated global tilapia production was 4.95 million metric tons (estimated value, $10.3 billion), with the People’s Republic of China, Indonesia, and Egypt being the three largest producers (see http://www.fao.org/fishery/statistics/global-aquaculture-production/en). Since 2014, strains of tilapia lake virus (TiLV) have spread globally, causing 10 to 90% mortality in cultured tilapia (Oreochromis spp. and hybrids) fry, juveniles, and adults in Colombia (1), Ecuador (2), Egypt (3), India (4), Indonesia (5), Israel (6), Malaysia (7), the Philippines (8), Peru (9), Tanzania (10), Thailand (11), Taiwan Province of China (12), and Uganda (13). Clinical signs associated with TiLV infections include lethargy, anorexia, and swimming at the surface away from schooling tankmates. Infected fish display gross lesions, including gill pallor, exophthalmia, body discoloration (darkening), scale protrusion and loss, and ascites (2, 13, 14). The most common microscopic lesions associated with TiLV infections include hepatitis and encephalitis lesions (15).

Herein, we present the complete genome sequence of a TiLV isolate (WVL18053-01A) isolated during a diagnostic investigation of a 2018 mortality event involving juvenile Nile tilapia (Oreochromis niloticus) in a Thai aquaculture facility. The virus was isolated from a pooled brain tissue homogenate inoculated onto a confluent monolayer of the striped snakehead cell line (SSN-1; E-11 subclone) maintained in Leibovitz medium (L-15; GE Healthcare Life Sciences) supplemented with 2% fetal bovine serum (Gibco) at 25°C. RNA was extracted from the SSN-1 culture supernatant using a QIAamp viral RNA minikit (Qiagen) according to the manufacturer’s instruction. A cDNA library was generated using a NEBNext Ultra RNA library prep kit (New England Biolabs) and sequenced on an Illumina MiSeq sequencer. *De novo* assembly of the paired-end reads performed in SPAdes version 3.10.0 with default parameters (16) recovered all 10 segments of the genome, and the average coverage ranged between 3,866 and 22,382 reads/nucleotide. BLASTN analysis of segment 1, which encodes the PB1 subunit, revealed the highest identity (99.1% [1,546/1,560 nucleotides]) to TiLV strain CL (accession no. KY615742) sequenced from diseased tilapia cultured in Thailand.

Although the socioeconomic impacts of TiLV have not yet been fully realized, it is believed to be a contributing factor to the “summer mortality” syndrome, resulting in production losses of 98,000 metric tons of Nile tilapia cultured in Egyptian fish farms worth approximately $100 million (17). It has also been suggested that TiLV may have significantly impacted wild tilapia stocks and the biodiversity in the Sea of Galilee in Israel (14). To date, TiLV has not been detected in North American tilapia stocks. Validated diagnostic assays and a coordinated North American surveillance effort are needed to protect the naive North American tilapia industry from this devastating globally emerging virus.

### Accession number(s).

The genome sequences for TiLV isolate WVL18053-01A, obtained from Thailand, have been deposited in GenBank under accession no. MH319378 to MH319387.
